# Root plasticity under abiotic stress

**DOI:** 10.1093/plphys/kiab392

**Published:** 2021-08-30

**Authors:** Rumyana Karlova, Damian Boer, Scott Hayes, Christa Testerink

**Affiliations:** Laboratory of Plant Physiology, Wageningen University, 6700 AA Wageningen, The Netherlands

## Abstract

Abiotic stresses increasingly threaten existing ecological and agricultural systems across the globe. Plant roots perceive these stresses in the soil and adapt their architecture accordingly. This review provides insights into recent discoveries showing the importance of root system architecture (RSA) and plasticity for the survival and development of plants under heat, cold, drought, salt, and flooding stress. In addition, we review the molecular regulation and hormonal pathways involved in controlling RSA plasticity, main root growth, branching and lateral root growth, root hair development, and formation of adventitious roots. Several stresses affect root anatomy by causing aerenchyma formation, lignin and suberin deposition, and Casparian strip modulation. Roots can also actively grow toward favorable soil conditions and avoid environments detrimental to their development. Recent advances in understanding the cellular mechanisms behind these different root tropisms are discussed. Understanding root plasticity will be instrumental for the development of crops that are resilient in the face of abiotic stress.

## Teaser

Recent discoveries show the importance of root system architecture plasticity for the survival and growth of plants under several abiotic stresses.

## General introduction

Climate change alters rainfall patterns and temperature, forcing ecological and agricultural systems to shift, transform or collapse (Raza et al., 2019; Chen and Gong, 2021). These changes in the environment, coupled with intensive agriculture, lead to soil degradation. Desertification (Huang et al., 2020) and salinization (Harper et al., 2021) are expected to increase in the future, threatening crop production, food security, and plant biodiversity. Soil degradation and various other abiotic stresses severely affect plant growth and account for the vast majority of global loss of crop yield (Teh and Koh, 2016). Considering the projected increase of the global human population and the increasing demand for animal protein consumption as well as the severity of present and future abiotic stresses, a major challenge will be to preserve biodiversity while sustainably feeding the global population. One approach to close the yield gap in the future is to study the adaptative abilities of different crops to abiotic stresses (Raza et al., 2019). These abilities are key to breed for more resilient crops that have the potential to counter soil degenerative processes, mitigate climate change, and produce stable yields (Duarte et al., 2013; Hasanuzzaman et al., 2014; Pessarakli and McMillan, 2014).

**Advances**

- Osmotic stress regulates xylem patterning and induces suberin formation. DEEPER ROOTING 1 (DRO1) increases the gravitropism of rice roots and promotes drought tolerance.
- Salt stress reduces the root apical zone size and increases the basal zone size of tomato plants grown in soil rhizotrons, in Arabidopsis *CYP79B2* and *HKT1* are associated with lateral root growth maintenance under salt stress.
- ABA is required for root growth and hydrotropism in drought conditions, while halotropism is dependent on auxin redistribution and internal Na^+^/K^+^ balance.
- Formation of ROL in roots is an important trait for flooding tolerance in plants.
- Root responses to warm temperatures require brassinosteroid and auxin signaling.

Plant roots provide anchorage, uptake, storage and transport of minerals and water. Plants can communicate and interact with the soil microbiome and other plants via their roots (Bao et al., 2014; Koevoets et al., 2016; Ryan et al., 2016; Anten and Chen, 2021). Roots show high developmental plasticity and often adapt to their environment. The spatiotemporal configurations of roots are referred to as their root system architecture (RSA). RSA has been defined as the geometric description of the shape (topology and distribution) of the root system (Lynch, 1995). In this review, we include RSA traits such as root positioning, length, angle, branching, surface area (including root hairs), coverage, and diameter. Studying the RSA of crops can provide insights into the genetic background of root traits for abiotic stress tolerance (Koevoets et al., 2016). This knowledge can be applied to new breeding strategies for stress-tolerant crops with stable yields even in challenging environments. Moreover, understanding RSA plasticity can give insights into the adaptability of plant species, allowing for novel strategies for replanting plants with adapted root architecture in places with extensive soil degradation to revert this process and support ecological succession. This review is aimed at highlighting recent discoveries on the molecular and cellular mechanisms behind root developmental plasticity as well as its importance for the survival of plants under abiotic stresses.

## Drought and salt

Drought and salt are some of the most widespread abiotic stresses for plants. In addition to drought caused by water deficit, water deficit may occur in saline and cold soils and even flooded soils; all conditions that limit plant water uptake (Salehi-Lisar and Bakhshayeshan-Agdam, 2016; Takahashi et al., 2020). Drought directly hampers root growth and development (Comas et al., 2013; Gupta et al., 2020), and low water uptake by the roots puts extra tension on xylem tissue. Without acclimation of the xylem, this may cause embolism of the xylem resulting in hydraulic failure (Sevanto, 2014; Levionnois et al., 2020; Li et al., 2021). Drought stress also affects nutrient uptake as nutrient mobility and diffusion is hampered (Rouphael et al., 2012; He and Dijkstra, 2014). It alters soil microbial populations and activity (Naylor and Coleman-Derr, 2018; Nguyen et al., 2018) and reduces the penetrability for the root systems, adding additional abiotic stress, soil compaction (Correa et al., 2019). Finally, postdrought rewatering of plants may provoke additional oxidative stress (Bian and Jiang, 2009).

Similar to drought, soil salinity provokes water deficit and nutrient imbalance. The low water potential of saline soils makes it difficult for roots to take up water by osmosis and salt ions compete with enzymes involved in the uptake and translocation of essential nutrients within plants (Van Zelm et al., 2020). Studies have shown that supplementation of plants with micronutrients (alone or in form of biostimulants) may help to alleviate salt stress, suggesting that micronutrients may be a limiting factor to regular growth and development in saline soils (Ghasemi et al., 2014; Torabian et al., 2018; Campobenedetto et al., 2021). Salt, like drought stress, also induce changes in the soil microbial populations (Qin et al., 2016; Rath et al., 2019).

In addition to water and nutrient deficit, soil salinity also provides (ion) toxicity stress. Accumulation of salt ions leads to hampered cell cycles and cytotoxic effects (West et al., 2004). Although most toxicity effects are attributed to sodium, chloride ions can also be detrimental by inducing chlorosis, chlorophyll deficiency that hampers photosynthesis (Tavakkoli et al., 2011). Drought and salinity stress perception have been suggested to occur through osmotic signals, cell wall integrity, plasma membrane lipids and ROS sensors (Lamers et al., 2020). In roots and shoots, the most well-described signal provoked by drought is the plant hormone abscisic acid (ABA) (Takahashi et al., 2020). In roots, ABA signaling coupled with modulation of auxin biosynthesis and transport appears to mediate changes in root architecture, morphology and anatomy to minimize water loss and maximize water uptake (Korver et al., 2018; Lamers et al., 2020; Li et al., 2021).

## Tropisms and root branching

### Responses to water deficit

**Figure 1:**
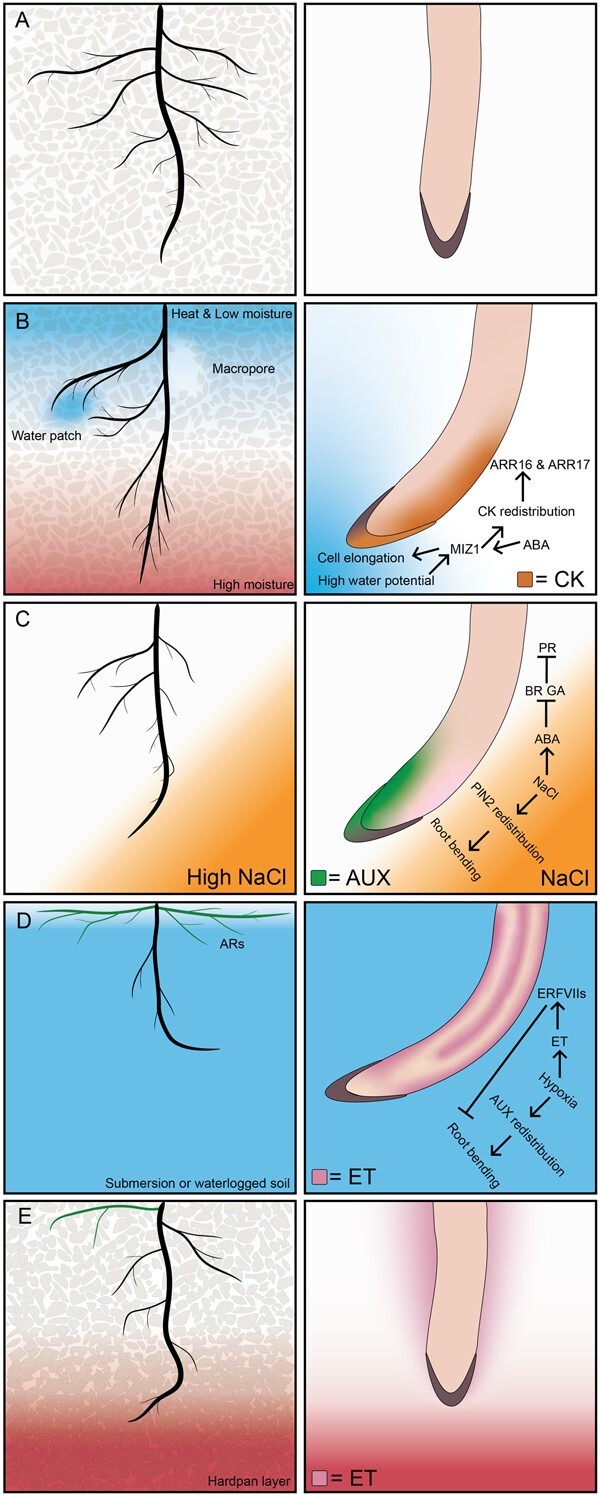
Plant RSA responses to abiotic stresses and their molecular regulation. A, Control: RSA under conditions with minimal abiotic stresses. B, Drought and heat: root growth and expansion in deeper soil layers, caused by an increase in gravitropic response and elongation of the roots. Local high moisture patches are sought through active growth toward water gradients known as positive hydrotropism. Moreover, root systems branch extensively in these areas by a process called hydropatterning. The side of the root with lower water potential accumulates cytokinin (CK), stimulating cell division asymmetrically, thereby allowing curvature of the root. CK accumulates at the site of the root with low water content and induces the expression of ARR16 and ARR17, which activate asymmetric cell division, resulting in bending of the roots. ABA can also induce the expression of MIZ1 and regulate hydrotropism. The bending of to root toward water is induced in the elongation zone where SnRK2.2 and MIZ1 regulate the differential growth response. C, Salt: depending on the severity of the salt concentration, salt type and plant sensitivity, root systems show positive or negative halotropism. Salt tolerant species, known as halophytes grow toward mild salt concentrations (positive halotropism), while most salt-sensitive plants, known as glycophytes, display negative halotropism. The gravitropic response is repressed. Salt stress induces ABA accumulation in the root tip, which inhibits GA and BR signaling and meristem size and PR elongation as well as reducing local auxin (AUX) levels. Salt stress induces PIN2 internalization and redistribution at one side of the root, causing differential auxin accumulation (green) and bending of the root away from the salt stress through negative halotropism. D, Flooding: root systems of plants respond to hypoxia by halting root growth, and stimulating ARs that grow sidewards to increase chances of improved oxygen uptake. The PR tip gravitropic response is inhibited leading to more lateral growth. This phenomenon of growth toward oxygen gradients is driven by ethylene (ET) signaling and has been referred to as aerotropism. Auxin regulates AR emergence by stimulating ethylene synthesis genes. Hypoxia induces auxin polar redistribution which leads to root bending (opposite of gravity) toward the soil surface. Hypoxia together with ethylene also induces the expression of ERFVII TFs that in turn can inhibit root bending. E, Soil compaction. For many plants, it remains unknown how plants respond to soil compaction, which is induced by agricultural practices and land management. Soil compaction leads to hypoxia, mimicking flooding stress; and leads to stimulation of ARs. Moreover, ethylene signaling represses root growth as well as the gravitropic response, thought to increase the tortuosity (curving nature) of the roots to increase maneuverability toward local cracks, soil pores, and less dense soils. Note that for water and salt gradients the image presents a directional response while in the case of submergence and compaction the root angle reflects repression of gravitropic response and can be in either direction.

Under mild stress, architectural adaptations are tailored toward *drought or salt avoidance* (Figure 1). Roots grow toward areas of higher water availability, often away from the dry top-soil layers where heat and salinity stress are most severe (Comas et al., 2013; Gandullo et al., 2021). Drought generally induces a *parsimonious* root architecture (Lynch, 2013, 2018) with fewer axial/lateral roots and a generally deeper rooting structure (Zhan et al., 2015; Dinneny, 2019). Directional root growth toward areas of higher water is realized by investment into an elongation of roots while increasing the gravitropism response—adjusting root angles downward (Uga et al., 2013). Deeper rooting allows for efficient water capture and thereby ameliorates drought stress. Root angle is controlled by columella cells, which contain amyloplasts that sediment in the direction of gravity in the root tips. The asymmetric distribution of amyloplasts directs auxin flow to the lower side of the root. Relatively low auxin levels on the upper side of the root allow for increased elongation and the downward bending of the main root (Ge and Chen, 2019). Lateral roots, crown roots, and adventitious roots (ARs) tend to grow less toward gravity. Recent insights indicate that cytokinin functions as a potent repressor of gravitropic response in these root types (Waidmann and Kleine‐Vehn, 2020).

Uga et al. (2013) showed that certain alleles of *DEEPER ROOTING 1* (*DRO1*) and its homologs increase the gravitropism of rice (*Oryza sativa* L.) roots, effectively benefitting drought stress avoidance (Kitomi et al., 2020). Both DRO1 and qSOR1 (quantitative trait locus for SOIL SURFACE ROOTING 1), belonging to the IGT gene family (named after their IGT domain), are negatively regulated by auxin (Waite and Dardick, 2021). Although their molecular function is unclear at present, it has been suggested that DRO1 and qSOR1 may have a role in establishing auxin gradients in the root tips (Waite et al., 2020). *DRO1* homologs have been identified in several plant families including Arabidopsis and *Prunus*, and *DRO1* clearly promotes deeper rooting and lateral root angle in these species (Guseman et al., 2017).

Positive hydrotropism, the bending of roots toward patches of water is another factor that influences RSA and drought tolerance (Dietrich et al., 2017; Dinneny, 2019). ABA was shown to be involved in the hydrotropism response of roots (Dinneny, 2019). ABA signaling subclass III Snf1-related kinases (SnRK2s) as well as MIZU KUSSEI1 (MIZ1) specifically expressed in the root cortex of the elongation zone are required for hydrotropism (Dietrich et al., 2017). Heterogeneous water presence at the root tip allows MIZ1 to generate a Ca^2+^ signal (Shkolnik et al., 2018; Tanaka-Takada et al., 2019). The Ca^2+^ wave travels through the phloem to the elongation zone, at which point Ca^2+^ becomes asymmetrically distributed according to the water gradient. Interestingly cytokinin is also asymmetrically distributed during the hydrotropism response (Chang et al., 2019; Figure 1). MIZ1 is also required for the hydrotropism-related asymmetric cytokinin redistribution. Furthermore, low ABA concentrations can induce root growth and promote hydrotropism by inhibiting the activity of PP2C phosphatases and enhancing the root apoplastic H^+^ efflux via H^+^-ATPase2 in both salt and drought conditions (Miao et al., 2021).

Another drought-avoidance strategy involves the control of lateral root emergence under drought through *hydropatterning* and *xerobranching*. Hydropatterning is the preferential branching of roots into areas with high water content (Bao et al., 2014; Orosa-Puente et al., 2018). Hydropatterning is independent of the ABA signaling (Bao et al., 2014), but it is dependent on AUXIN RESPONSE FACTOR 7 (ARF7). ARF7 is SUMOylated specifically on the side of the root in contact with a dry environment (Orosa-Puente et al., 2018). SUMOylation of ARF7 enhances its interaction with its repressor, IAA3 (indole-3-acetic acid). Inhibition of ARF7 by IAA3 prevents the expression of lateral root initiation genes and lateral root initiation. *Xerobranching* is the strong repression of lateral root formation under drought when the roots are in aerial pores in the soil (Orman-Ligeza et al., 2018). When roots lack water contact in such soil macropores, ABA accumulates. The ABA binds to signaling PYR/PYL receptors, resulting in local auxin decrease. The ABA-induced auxin reduction could lead to the inhibition of lateral root formation (Orman-Ligeza et al., 2018; Bloch et al., 2019). However, whether ABA signaling also plays a role in lateral root formation during xerobranching remains unknown.

### Coping with salinity

A marked response of the main root influencing root architecture in saline soils is halotropism (Figure 1). In roots of salt-sensitive, glycophyte species (Van Zelm et al., 2020), including Arabidopsis, tomato and Sorghum, negative halotropism (root bending away from salty environments) is a key strategy to avoid salt stress. Halotropism is dependent on auxin redistribution in the root tip (Galvan-Ampudia et al., 2013; van den Berg et al., 2016). The plasma membrane auxin transporter PIN2 relocalizes in a polar manner upon encountering salt. PIN2 promotes a relative reduction in auxin levels on the saline side of the root and enhanced bending away from salinity. PIN2 relocalization is associated with clathrin-mediated endocytosis and controlled by phospholipase PLDζ2 (Galvan-Ampudia et al., 2013; Korver et al., 2020). A Genome-Wide Association Study (GWAS) into natural variation of halotropism within Arabidopsis identified AtDOB1 and AtCHX13 as additional players in the response (Deolu-Ajayi et al., 2019). Both genes are upregulated under salt stress, and both appear to be involved in Na^+^/K^+^ homeostasis, suggesting that internal ion homeostasis is likely a key factor underlying the halotropism response (Galvan-Ampudia et al., 2013; Pierik and Testerink, 2014; Szepesi, 2020). Interestingly, several salt-tolerant, halophyte species such as *Brassica indica* and *Limonium bicolor*, show positive halotropism in heterogenous mild saline soils (Shelef et al., 2010; Leng et al., 2019). It remains unknown whether similar molecular mechanisms regulate halotropism in the roots of these species.

When challenged with high salt stress, the growth of primary and lateral roots is arrested. This arrest shows interesting temporal dynamics. Both root types first enter a quiescent phase (QP) (Geng et al., 2013; Julkowska and Testerink, 2015; Van Zelm et al., 2020). In Arabidopsis, the QP generally lasts longer in lateral roots than primary roots (PRs) (Duan et al., 2013) and is established by ABA-dependent (Zhao et al., 2014) inhibition of gibberellic acid (GA) and brassinosteroid (BR) signaling (Geng et al., 2013). Following the QP (lasting hours to several days), is a recovery phase with increased GA, BR and JA signaling and reduced ABA signaling, and this leads to a partial recovery of growth during salt stress (Geng et al., 2013). This partial growth recovery is likely due to the decrease of root meristem size (Duan et al., 2013). Upon salt stress, ROS accumulation is linked to the programmed cell death at vital meristematic tissues in the root tips (West et al., 2004) and it is thought that this acts to protect the quiescent center from damage. A large-scale natural variation study in Arabidopsis (Julkowska et al., 2017), indicated that salt stress increases the variance in primary and lateral root growth between accessions. Subsequent GWAS studies indicated that natural variation in *CYP79B2* and *HKT1* (high-affinity Potassium Transporter 1) is associated with lateral root growth maintenance under salt stress. HKT1 is a sodium transporter that had been previously associated with salt tolerance in several plant species (Moller et al., 2009; Munns et al., 2012; Ali et al., 2019). While high *HKT1* expression is generally considered positive for salt stress tolerance in crops, it can be detrimental to root growth in young seedlings, due to the accumulation of toxic Na^+^ ions in their roots. *CYP79B2* on the other hand promotes the biosynthesis of IAOx, a precursor of the plant hormone auxin (IAA; Korver et al., 2018). *CYP79B2* is expressed in root zones where lateral roots emerge, and mutant lines lacking both *CYP79B2* and its homolog *CYP79B3* have shorter and less dense lateral roots, specifically under high salt conditions (Julkowska et al., 2017).

Defining a salt tolerance root ideotype is difficult. In a study with a limited set of Arabidopsis accessions, root systems with many, short lateral roots resulted in a lower internal shoot Na^+^/K^+^ ratio (Julkowska et al., 2014). In a more natural soil-rhizotron setup, salt stress was shown to significantly reduce the root apical zone size and strongly increased the basal zone size of tomato, resulting in the placement of lateral roots in the deeper zones of the soil with lower salt accumulation (Gandullo et al., 2021). Interestingly, a positive association between the root branched zone and the root K^+^/Na^+^ ratio was observed. In soil conditions, it is likely that the best adapted root system is the one that efficiently avoids saline environments by both root direction and placement and outgrowth of lateral roots. On the other hand, (Kitomi et al., 2020) showed that a shallower root growth angle could be beneficial for salt tolerance in rice based on the qSOR1 loss-of-function mutants. Studying the molecular and cellular basis behind these changes may provide further insights into how plants cope with heterogeneous salt environments.

### Root anatomical changes

Drought and salt induce also anatomical changes in the roots (Klein et al., 2020; Van Zelm et al., 2020). For example under drought, cortical tissue with fewer, but large cell files is beneficial anatomy conferring tolerance (Chimungu et al., 2014; Colombi et al., 2019). Cortical tissues can be converted into porous aerenchyma tissue (Lee et al., 2016; Klein et al., 2020) in which cortical cells are lysed, creating an intercellular cell-less space that requires little energy to maintain. This process also releases nutrients to the surrounding cells and is prevalent in older roots that are no longer taking up water efficiently. The formation of lysigenous aerenchyma is also stimulated under salt stress (Silva et al., 2021).

Under both drought and salt stress, the biosynthesis and deposition of suberin is stimulated in PRs, in particular in the endodermis and exodermis layers where suberin limits radial water loss (Lynch, 2018; Van Zelm et al., 2020; Siddiqui et al., 2021). Studies in the grape show that generally enhanced suberin biosynthesis of the root system are associated with increased drought tolerance (Yıldırım et al., 2018), while suberin layers in *fine* roots (roots ≤ 2 mm) may increase susceptibility to drought. The benefit of suberin may therefore be root-type specific (Yıldırım et al., 2018; Cuneo et al., 2020). Studies comparing wild barley with domesticated cultivars indicate that wild barley has more suberin deposition in the exodermis under drought, similar to other drought-adapted species (Kreszies et al., 2020; Yang et al., 2020). Suberin was shown to function in salt exclusion, as a barrier in the endodermis (Ranathunge and Schreiber, 2011; Barberon et al., 2016). The suberin deficient mutant cyp86a1 is salt-sensitive compared with the wild-type and accumulates more Na^+^ ions (Wang et al., 2020a). The most well-described TFs controlling suberization belong to the MYB family (Baldoni et al., 2015; Zhang et al., 2020a). *MYB41* is upregulated by drought stress, salt stress and ABA, and stimulates suberin biosynthesis and deposition in Arabidopsis and grapevine. MYB41 regulates the expression of genes associated with the biosynthesis pathways of suberin and other waxy compounds that prevent water loss during salt stress (Kosma et al., 2014). Another salt stress-responding MYB TF, SUBERMAN (MYB39) enhances suberin deposition in the endodermis (Cohen et al., 2020). Other salt/drought stress induced TFs such as NACs (Jeong et al., 2013; Dudhate et al., 2021) and WRKYs (Krishnamurthy et al., 2020) have also been shown to stimulate the suberin biosynthesis pathway. ABA appears to promote suberization whereas ethylene represses this process (Barberon et al., 2016).

Lignin deposition is widely reported to enhance drought tolerance by forming a water-resistant barrier around mature xylem tissue (Xu et al., 2017; Liu et al., 2018; Sharma et al., 2020). Interestingly, mutants overexpressing lignin biosynthesis genes often additionally show longer PRs (Li et al., 2020; Xu et al., 2020). Like suberin, lignin biosynthesis is regulated by MYB TFs (Baldoni et al., 2015). During drought stress, xylem vessel size was shown to increase, and these large xylem vessels are responsible for increased root conductivity (Tan et al., 2020). Such root xylem distributions have been proposed to provide high hydraulic conductance while reducing the risk of hydraulic failure (Li et al., 2021). Large xylem cells with high conductivity can allow for deeper rooting (Strock et al., 2020), but under prolonged stress, most drought-tolerant crops opt for numerous small xylem vessels (Klein et al., 2020; Ramachandran et al., 2020; Strock et al., 2020), which can have big consequences for plant survival under drought (Scoffoni et al., 2017; Levionnois et al., 2020). In poplar salt stress resulted in reduced xylem cells and vessel diameters (Junghans et al., 2006), while in tomato roots lignified xylem cells increased under salt stress (Sánchez-Aguayo et al., 2004). In Arabidopsis, Shinohara et al., (2019) showed that in a thermospermine-deficient mutant salt hypersensitivity is linked to excessive xylem development, which suggests an opposite effect of salt compared with drought stress on xylem formation.

The development of the xylem in the roots is controlled also by ABA. Drought-induced ABA accumulation activates microRNAs 165 (miR165) and miR166 to repress class III HD-ZIP TFs. Class III HD-ZIPs repress xylem formation and so their inhibition leads to the additional proto-xylem formation during drought stress (Ramachandran et al., 2018).

In olive trees, salt induces thickening of the high suberin cork layer in roots. This cork (periderm) layer strongly accumulates salts and reduces salt levels in the inner, salt-susceptible layers of the roots (Campilho et al., 2020; Tan et al., 2020). Many species have the ability and the molecular framework to develop periderm tissue (Wunderling et al., 2018). Yet, little is known about the potential contribution of the periderm to abiotic stresses such as salt in non-woody plants. In addition to promoting root thickening, salt also accelerates root differentiation (Byrt et al., 2018). In salt-treated plants, the formation of the endodermis, Casparian strip, and exodermis layers starts closer to the root tip (Davis et al., 2014; Van Zelm et al., 2020).

### Root hair formation in salt and drought

In addition to the adjustment of primary and lateral roots, recent research indicates the importance of root hair growth and development under drought and salt. Enhancement of root hair length and density has been reported to be a key factor in conferring drought tolerance in crops (Cheng et al., 2016; Zhang et al., 2020b). Root hairs enhance the root surface area (Segal et al., 2008; Lynch, 2018) and may mediate higher penetrability on harder substrates (reviewed by Salazar-Henao et al., 2016). Several genes influencing root hair formation under droughts such as *EXPB7* (He et al., 2015) and *WOX11* (Cheng et al., 2016) also affect multiple traits conferring drought tolerance. The GLABRA2 TF negatively regulates root hair growth in response to osmotic stress (Wang et al., 2020b). Under salt stress, both root hair length and density were shown to be decreased (Wang et al., 2008; Robin et al., 2016). It has been proposed that the Salt Overly Sensitive (SOS) pathway is involved in salt-responsive root hair modulation. *sos1-3* lines show dramatically reduced root hair length or root hair density under salt (Wang et al., 2008). Overexpression of *Triptychon* (*TRY*) TFs from the halophyte *Limonium bicolor* in Arabidopsis (Leng et al., 2020) showed their involvement in the salt tolerance root hair development pathway.

## Heat and cold

For every species of plant, there is a range of temperatures at which growth is permitted. Temperatures above this range (heat stress) and temperatures below this range (cold stress) generally inhibit growth (McMichael and Burke, 1998). Thermomorphogenesis is the effect of ambient (mild) temperatures on plant morphology. In Arabidopsis, growth temperatures of around 12°C to 28°C are typically considered as thermomorphogenic. Ambient temperature perception in the shoot is relatively well understood (Hayes et al., 2020). Shoot thermomorphogenesis is controlled through the temperature-sensitive function of the phytochrome B photoreceptor. Given that phytochrome B requires light for its activation and the (mature) root is located underground, it is probable that other temperature sensors are utilized in the root. Recently, it was shown that the circadian clock component ELF3 also functions as a shoot temperature sensor (Jung et al., 2020). At warm temperatures, ELF3 undergoes phase separation to an inactive state. This process is presumably independent of light and so could conceptually play a role in root thermomorphogenesis. Another recent article showed a temperature signaling mechanism that requires the mitogen-activated protein kinase kinase kinase kinase4 (MAP4K4), TOT3. The TOT3 pathway is independent of phyB and also has the potential to act in the root (Vu et al., 2021). There appears to be a genetic linkage between shoot and root elongation at warm temperatures (Gaillochet et al., 2020), but dissected roots are also capable of responding to temperature cues (Bellstaedt et al., 2019). This suggests that root elongation at ambient warm temperatures may be governed both directly by signaling events in the root, and indirectly through signaling events in the shoot.

Outside of the ambient temperature range (during heat or cold stress), there are numerous potential sensors. Heat stress interferes with protein folding, ion channel activity, cell membrane integrity and enzyme function. All of these signals could conceivably contribute to root growth arrest during heat stress. In Arabidopsis shoots, sudden exposure to heat stress is associated with Ca^2+^ and ROS waves (Hayes et al., 2020). Ca^2+^ and ROS waves are observed in response to multiple environmental stresses, and it is feasible that they are involved in the inhibition of root growth under heat stress. Recently two cold sensing mechanisms were identified. In Arabidopsis shoots, mRNA translation rates drop dramatically on exposure to cold temperatures (Guillaume-Schöpfer et al., 2020). The inhibition of translation is coupled with an increase in intracellular free Ca^2+^. Intracellular Ca^2+^ activates CAMTA transcription factors and leads to the induction of cold-induced genes. Strikingly, chemical inhibition of translation has a similar effect on intracellular free Ca^2+^ and *CAMTA*-dependent gene expression. It has been proposed that cold temperatures reduce the translational efficiency of ribosomes and this promotes Ca^2+^ release and downstream signaling events (Guillaume-Schöpfer et al., 2020). This work was based on whole seedlings, but given that cold stress also provokes transient increases in intracellular Ca^2+^ in the root (Choi et al., 2014), a similar mechanism may play a role in cold stress-mediated root inhibition.

Another recent study found that reduced growth rates at cold temperatures can act as a signal itself (Zhao et al., 2020). NTL8 (a transcription factor that promotes vernalization) accumulates in cold grown roots, without changes in gene expression or protein stability. Modeling approaches demonstrated that reduced cell elongation at cool temperatures reduces the cellular dilution of NTL8 in the root tip. This conclusion was supported by the fact that several pharmacological agents that supress root growth also led to NTL8 accumulation. It is not clear whether NTL8 accumulation plays a role in repressing root elongation at cool temperatures, but the same concept would likely hold for any long-lived protein. Moreover, it’s feasible that a similar mechanism contributes to root morphology under any stress that reduces root elongation.

## The effect of temperature on root architecture

At cool temperatures (around 12°C–20°C) Arabidopsis roots develop a compact structure, whereas at warm temperatures (around 21°C–28°C) roots increasingly adopt an elongated, open architecture. Both heat and cold stress generally inhibit root elongation, but in field conditions, they are unlikely to result in the same root architecture. Temperature extremes are normally preceded by warm or cool periods through which root growth is permitted. A heat-stressed root is, therefore, more likely to have an elongated structure and cold stressed root is more likely to be compact.

Warm ambient temperatures promote primary/seminal root elongation in diverse monocots and dicots (Al-Ani and Hay, 1983; McMichael and Burke, 1998; Yang et al., 2017; Figure 1). In Arabidopsis and maize, warm temperature increases cell elongation rates in the root elongation zone (Pahlavanian and Silk, 1988; Nagel et al., 2009; Yang et al., 2017), and reduces root meristem size (Nagel et al., 2009; Martins et al., 2017; Yang et al., 2017). Arabidopsis main roots are slightly thinner at warm temperatures (Yang et al., 2017), but in maize, the opposite trend has been observed (Pahlavanian and Silk, 1988). Warm temperature promotes lateral root development in many species (McMichael and Burke, 1998; Nagel et al., 2009; Wang et al., 2016), but its effect on lateral root elongation is species-specific. Lateral root elongation was enhanced at warm temperatures in cotton and sunflower, but not affected in maize (McMichael and Burke, 1998; Nagel et al., 2009; Waidmann et al., 2020). In soybean and oilseed rape, warm temperatures increased the angle between primary and lateral roots, resulting in a more open structure (Kaspar et al., 1981; Nagel et al., 2009). In Arabidopsis, warm temperatures promoted lateral root gravitropism, resulting in a deeper and more vertically oriented root system (Rellan-Alvarez et al., 2015).

The adaptive benefit of RSA changes at warm temperature effects is an open question. There is a negative correlation between temperature and water availability (Livneh and Hoerling, 2016). High temperature increases evaporation from soils and evapotranspiration through plants. The reduced transpiration of plants during drought stress may induce also heat stress in the leaves (Lamaoui et al., 2018). It may be that the elongated structure adopted by roots at warm temperature serves to enhance water uptake (Uga et al., 2013). Intriguingly, mild drought has a similar effect on root architecture as ambient warm temperatures (Rellan-Alvarez et al., 2015). It has even been postulated, that temperature sensing in the roots could have derived from a drought sensing pathway (Ludwig et al., 2021), although experimental evidence of this is currently lacking.

Temperature extremes are predicted to become more common in the future and optimizing the RSA of crops may help to increase their heat tolerance. A recent study on the temperature-stress resilience of plants on a global scale found that (as with animals) there is more variation in the ability of plants to survive cold stress than heat stress (Lancaster and Humphreys, 2020). The authors found that there is much more variation in cold stress tolerance than warm stress tolerance in plants. This suggests that there are many different pathways that plants can acquire cold tolerance, but that the development of heat tolerance is more difficult. Breeding plants for heat tolerance may therefore present a sizable challenge.

### Temperature signaling in the root

PR elongation in Arabidopsis is the most well-characterized root response to ambient warm temperature. Even so, there is still only limited information on how this developmental process is regulated. Warm temperatures promote auxin signaling at the root tip (Zhu et al., 2015; Wang et al., 2016; Feraru et al., 2019; Sun et al., 2020). It appears that brassinosteroid signaling is also involved, but its directionality is debated. Some evidence implies that brassinosteroid signaling is reduced at warm temperature (Martins et al., 2017), whereas evidence from other studies implies that brassinosteroid signaling is increased (Sun et al., 2020). These conflicting results could be explained by the tissue-specific nature of brassinosteroid signaling. In the epidermis, brassinosteroid promotes PR elongation, whereas in the stele brassinosteroid represses elongation (Vragovic et al., 2015). Investigation of the tissue-specific effects of temperature on brassinosteroid signaling may help to resolve this point. Warm temperatures promote the transcription of heat shock protein (*HSP*) chaperones. HSP90.1 has been shown to promote the stability of the auxin receptor TIR1 (Wang et al., 2016) and the negative regulator of brassinosteroid signaling BIN2 (Samakovli et al., 2014) and so *HSP90.1* probably contributes to ambient temperature signaling in the root.

Currently, very little is known about how warm temperatures promote lateral root development. It is feasible that (as in the PR tip) increased auxin signaling is required. Lateral root initiation requires the rephasing of the circadian clock (Voss et al., 2015) and so the warm temperature-mediated inactivation of clock-component ELF3 could well play a role. To our knowledge, there are no studies on the mechanism of warm temperature-mediated inhibition of root gravitropism. Further investigation into this phenotype may yield interesting insights into the control of root thermomorphogenesis.

## Flooding

Flooding is the collective term of two distinct abiotic stresses; soil waterlogging and submergence. Flooding has a multitude of detrimental effects on plant growth and development (reviewed by Sauter, 2013). One of the predominant stress factors in a flooded environment is the inhibition of gas diffusion, leading to oxygen deficiency (hypoxia). Hypoxia hampers respiration and limits energy production. Reduced energy production leads to reduced uptake of nutrients, (Martínez-Alcántara et al., 2012) and water, and metabolic imbalance. Hypoxia also inhibits root hydraulic conductivity, restricting water uptake despite environmental excess. Following flooding, re-exposure of plants to oxygen additionally results in oxidative damage (Tamang and Fukao, 2015; Yuan et al., 2017; Yeung et al., 2018; Da-Silva and do Amarante, 2020).

One of the first signals of hypoxia is the rapid accumulation of ethylene around the roots, due to reduced gas diffusion. The accumulation of ethylene acts to promote root meristem hypoxia tolerance (Sasidharan and Voesenek, 2015; Hartman et al., 2019). During postflooding recovery the hormone jasmonate (JA) accumulates rapidly in Arabidopsis rosettes. The transcription factor MYC2 is upregulated upon JA accumulation, which in turn stimulates genes involved in antioxidant synthesis pathways (Yuan et al., 2017). JA-mediated antioxidant synthesis likely limits oxidative damage in these conditions (Yuan et al., 2017; Yeung et al., 2018). Curiously, during post-flooding recovery, ethylene seems to act as a negative regulator of the recovery by enhancing chlorophyll breakdown, water loss and senescence (Yeung et al., 2018). It is currently unclear if these signaling pathways in rosettes also apply to the root. Considering that the JA has a well-established role in defense against biotic stress, it is likely that the reoxygenation response overlaps with known biotic defense responses (Zhou et al., 2019).

### Root angle and branching during flooding

The majority of RSA adaptations during flooding are directly related to maximizing oxygen uptake, controlling oxygen loss (Sauter, 2013). The first response to hypoxia includes a stop in both the formation and elongation of lateral roots (Shukla et al., 2019). These responses are driven by ethylene signaling (Sauter, 2013; Yamauchi et al., 2018; Lamers et al., 2020). The formation of lateral roots is repressed by ERF-TFs. RAP2.12 and HRE2 have been shown to bind and repress the expression of genes involved in lateral root primordia formation, such as *LBD16*, interfering with local auxin signaling that promote lateral root formation (Shukla et al., 2019).

Under flooding stress, plants also invest in ARs formation. ARs are roots that are formed post-embryonically from organs other than the root, such as the stem. Although certain plants can form ARs constitutively, de novo AR formation is particularly stimulated under flooding (Eysholdt‐Derzsó and Sauter, 2019). AR formation and elongation allows the root system to grow toward oxygen-rich surfaces or even the atmosphere (aerial AR), as AR growth angles differ from regular root types (Eysholdt‐Derzsó and Sauter, 2019). In rice, ARs have been reported to grow toward the surface (Lin and Sauter, 2018, 2019) enabling ethylene venting and re-aeration (Figure 1). Polar auxin redistribution by PIN1 and PIN2 in the root tip of ARs are involved in this process (Lin and Sauter, 2019). Moreover, ARs provide additional anchorage post-flooding and benefit nutrient uptake (Zhang et al., 2017; Eysholdt‐Derzsó and Sauter, 2019). Anatomically, flood induced ARs are relatively cost-efficient due to their low density of energy-demanding cells; they contain more aerenchyma, which improves internal aeration within roots (Zhang et al., 2017). In extreme cases of flooding, a plant’s original root system may be entirely substituted for an AR root system (Calvo-Polanco et al., 2012). Studies in flood-tolerant species indicate mechanistic variation underlying AR formation: ethylene has been reported to promote AR formation in rice (Lin and Sauter, 2018) while in Arabidopsis ethylene was found to inhibit AR formation (Veloccia et al., 2016). It is likely that due to evolutionary selection, flooding tolerant plants have evolved a different AR induction system. Plants can also alter their original roots angles and anatomy during flooding conditions. Hypoxic treatment induced Arabidopsis PR bending almost perpendicular to the gravity vector (Eysholdt-Derzsó and Sauter, 2017). After the re-establishment of normal oxygen conditions, the effect was reversed. In addition, it was shown that both RAP2.12 and HRE2 TFs negatively regulate hypoxia-driven root bending (Eysholdt-Derzsó and Sauter, 2017). Many questions remain to be answered regarding the root bending response, including the mechanism and adaptations that aquatic and wetland plants use in the regulation of hypoxia-driven root bending.

### Anatomical changes to flooding stress

The most well-described adaptations to flooding include the formation of aerenchyma and the formation of oxygen loss barriers (Vidoz et al., 2016; Yamauchi et al., 2018). The formation of aerenchyma is regulated by ethylene and high concentrations of ethylene stimulate ROS production in the root cortex which results in controlled programmed cell death (Yamauchi et al., 2011, 2018). As with AR formation, ethylene is involved in the induction of aerenchyma formation, but auxin signaling and transport is needed for constitutive aerenchyma formation in rice (Yamauchi et al., 2019, 2020). Plants have evolved root adaptations to inhibit oxygen loss during floods through the synthesis of physical barriers, Radial Oxygen Loss (ROL) barriers. Lai et al., (2012) showed that ROL at the root tips appears to be a positive trait that correlated with biomass and nutrient uptake across 35 plant species. ROL barriers are generally formed 20–30 mm behind the root tips and are characterized by an increase in suberization of epidermal root cells (Colmer et al., 2019). Importantly, ROL barriers which can be formed from the cortex layer to the rhizosphere (outer cell layers) of the root do not seem to hamper water or nutrient transport (Pedersen et al., 2021b). Besides ROL barriers preventing oxygen loss, they may also allow oxygen flow to root tips. Under anoxic conditions, anaerobic microorganisms secrete organic acids, many of which are phytotoxic, and hamper nutrient availability for plant roots. These toxic organic acids appear to be the major environmental stimulus for ROL barrier formation (Colmer et al., 2019). ROL barriers are predominantly reported to be formed in ARs of wetland plants (Kotula et al., 2017). Pedersen et al. (2021a) found ROL formation in lateral roots in teosinte (*Zea nicaraguensis*), a flooding-tolerant plant. In the future, the genetic background for this trait (Watanabe et al., 2017) might provide important insights on flood tolerance through ROL barrier development.

## Discussion and perspectives

In the recent years, many discoveries have been made concerning the molecular mechanisms and signaling of abiotic stresses perception in plants (Lamers et al., 2020). How abiotic stress perception impacts root plasticity is still an open question. Recently, several advances have been reported, including the discovery of DRO1 as a regulator of root angle, ABA controlled root growth, molecular mechanisms affecting halotropism and hydrotropism under salt and drought stress, ABA-mediated changes in xylem patterning during osmotic stress, the role of ROL barriers during flooding in teosinte and Glabra2- dependent root hair growth under drought and salt stress.

Some of the molecular regulations involved in the root tropisms under different stresses are less understood. An example is the interaction between ABA-dependent and cytokinin-dependent hydrotropism. It was shown that MIZ1 is induced by both ABA and cytokinin and thus could be the link between the two pathways regulating PR hydrotropism response. The role of ABA signaling in lateral root development during xerobranching needs to be addressed as well.

Furthermore, little is known about the molecular control of root thermomorphogenesis as well as on the mechanism of warm temperature-mediated inhibition of root gravitropism. In natural soils under drought stress, roots need to adapt their architecture and respond to drought, heat, and even soil compaction stresses at the same time. The links and molecular interaction between these soil stresses need further investigation.

So far only a handful of GWAS studies have been performed to identify root traits related to abiotic stress resilience. Moreover, this knowledge is restricted mainly to the model species Arabidopsis and performed *in vitro* and in controlled climate conditions. An interesting question is how the RSA plasticity of different crops contributes to their survival under abiotic stress conditions. For example, although tuber plants like potatoes are very important crops agronomically, research on the importance of their RSA for the resilience to abiotic stresses is limited. To identify whether the gene regulatory networks controlling root architecture under different abiotic stresses are conserved between species or translatable from the model plant Arabidopsis into crops species, it will be interesting to compare if the hydrotropism and other root tropism responses are regulated by the same molecular mechanisms and hormonal cross talks like shown for the model species Arabidopsis. The discovery of root traits in crops associated with resilience to abiotic stress(es) will lead to new breeding strategies and selected genotypes that can grow and produce a stable yield in less favorable or changing environmental conditions. These robust crops could grow better in salinized or dry soil with fewer nutrients which will help to close the yield gap in the future and reduce the use of freshwater resources. The recent emergence of molecular technologies including single-cell sequencing, CRISPR/CAS9 genome editing as well as tissue- and cell-specific promoters studies for imaging of cellular processes will greatly contribute to our understanding of crop root plasticity under stress (Shulse et al., 2019; Kajala et al., 2021; Lyzenga et al., 2021).

In the field, crops are grown in soil and experience different mild or severe stresses at the same time. However, little is known about how the soil quality, soil type and availability of nutrients and soil structure influences plant root traits (bending of the roots, root angle) and adaptations in crops (see Outstanding Questions). Recently it was shown that the growth of roots in compact soils is inhibited due to the accumulation of ethylene (Pandey et al., 2021), mirroring the pathway controlling hypocotyl emergence from compact soil (Shi et al., 2016). In addition, the root phenotype Multiseriate cortical sclerenchyma (MCS) associated with the ability to penetrate compacted soils was identified in maize and wheat. Interestingly MCS formation could be induced by exogenous ethylene (Schneider et al., 2021). Another potential root adaptive mechanism, root circumnutation (the ability of the root to undergo helical movement) and its molecular regulation were recently revealed (Taylor et al., 2021). Root circumnutation was proposed to serve as an adaptation of the PR to penetrate hard soils and to avoid obstacles in the soil. In rice, HK1 (histidine kinase-1 gene) was shown to be involved in the regulation of root circumnutation. Interestingly and in line with the proposed role of root circumnutation, the hk1 mutant is unable to explore/penetrate efficiently artificial solid surfaces or clay particles compared with the wild type rice roots.

Although it has been shown that abiotic stresses like drought and salt can change the rhizosphere community of the roots in different crops (Zhang et al., 2018; Hartman and Tringe, 2019), how RSA could influence the recruitment of beneficial root microbiome under abiotic stress remains to be discovered (see Outstanding questions). Another important direction of research is to understand the molecular mechanism of root-microbial interactions, the role of root exudates in these interactions in presence of abiotic stress or a combination of stresses. Future research should address the question of how crop RSA modulation interacts with the soil microbiome under stress. In summary, to understand the underlying mechanisms of plant root plasticity for the survival of crops under abiotic stresses, further research is needed to study root adaptations to single stresses in different environments (soil type, quality, and microbiome) and to multiple simultaneous stresses.

**Outstanding questions**

- How do plant roots recruit a beneficial rhizobiome under abiotic stress and what is the role of root exudates in this interaction?
- How do plants shape their root architecture under a combination of different stresses?
- Is there an interaction between soil type and root system plasticity of crops under abiotic stress?
- How does soil compaction affect the RSA of different crops?
- What is the contribution of the RSA plasticity of agronomically important tuber crops for their ability to survive under abiotic stress?
- What is the effect of abiotic stresses on alternative, less conventional plants not used in high input agroecosystems?

## Funding

This work was supported by the Dutch Research Council (NWO/OCW), as part of the MiCRop Consortium Programme, Harnessing the second genome of plants (grant number 024.004.014) and by NWO-TTW-H.I.P. (grant 16893 to C.T). We also acknowledge support by the European Union's Horizon 2020 Research and Innovation Programme under grant agreement No. 771134, ERA-NET Cofund SusCrop grant, being part of the Joint Programming Initiative on Agriculture, Food Security and Climate Change (FACCE-JPI) to C.T. and R.K., and a Wageningen Graduate Schools Grant/Award: Postdoctoral Talent Programme to S.H.

##  

*Conflict of interest statement*. The authors declare that there is no conflict of interest.

## Notes

All authors participated in writing the article.

The author responsible for distribution of materials integral to the findings presented in this article in accordance with the policy described in the Instructions for Authors (https://academic.oup.com/plphys/pages/general-instructions) is: Christa Testerink (christa.testerink@wur.nl).
